# ChIPprimersDB: a public repository of verified qPCR primers for chromatin immunoprecipitation (ChIP)

**DOI:** 10.1093/nar/gky813

**Published:** 2018-09-07

**Authors:** Stefan Kurtenbach, Rohit Reddy, J William Harbour

**Affiliations:** Bascom Palmer Eye Institute, Sylvester Comprehensive Cancer Center, and Interdisciplinary Stem Cell Institute, University of Miami Miller School of Medicine, Miami, FL 33136, USA; Bascom Palmer Eye Institute, Sylvester Comprehensive Cancer Center, and Interdisciplinary Stem Cell Institute, University of Miami Miller School of Medicine, Miami, FL 33136, USA; Bascom Palmer Eye Institute, Sylvester Comprehensive Cancer Center, and Interdisciplinary Stem Cell Institute, University of Miami Miller School of Medicine, Miami, FL 33136, USA

## Abstract

Chromatin immunoprecipitation (ChIP) has ushered in a new era of scientific discovery by allowing new insights into DNA-protein interactions. ChIP is used to quantify enriched genomic regions using qPCR, and more recently is combined with next generation sequencing (ChIP-seq) to obtain a genome wide profile of protein binding sites. Nevertheless, ChIP-qPCR remains an integral component of this technology for quality control purposes, before the library preparation and sequencing steps. In addition, ChIP-qPCR remains more time- and cost-effective for many focused projects in which the DNA regions of interest are already known. However, the DNA oligonucleotide primers needed for ChIP-qPCR are more challenging to design than for other qPCR projects. Here, we present the first public repository for ChIP oligonucleotides that have been verified to perform well in ChIP-qPCR experiments. ChIPprimersDB was developed by manual screening of publications to ensure primer quality and provide additional specific information on the ChIP experiments where the primers have been used. In addition to the primer sequences, the database includes information about the antibody, cells and tissues used in the experiment, information on the experimental design, and a direct link to the original publication. The database is linked at https://umiamihealth.org/bascom-palmer-eye-institute/research/laboratory-research/ocular-oncology-laboratory/chip-primers and hosted at https://www.chipprimers.com/.

## INTRODUCTION

Polymerase chain reaction (PCR) is widely used to amplify specific target DNA sequences in various applications. This targeted amplification is achieved by oligonucleotide primers flanking the sequence of interest that initiate the polymerase reaction. Since the invention of PCR in 1983 (1–3), the method has been widely adopted and modified to suit various purposes (4), including qPCR (5) and qRT-PCR (5,6), allowing the real-time quantification of PCR amplicons. Today, qPCR still remains the most sensitive technique for measuring minute quantities of nucleic acids in research and diagnostics applications.

The quality of PCR amplification is highly dependent on the specificity and efficiency of the primers. Specificity can be assessed by analyzing melting curves and separation of the PCR products on an agarose gel to verify the correct amplicon size, alongside Sanger sequencing. Primer efficiency is calculated by performing PCR with progressive template dilutions and is a measure of the amount of amplification per cycle. Numerous primer databases have been established to guide the selection of high quality primers (7–18). Further, publicly available algorithms allow the design of high quality qPCR primers that meet specific characteristics (19).

More recently, qPCR has been adopted to quantitate the enrichment of DNA fragments in chromatin immunoprecipitation (ChIP) experiments. ChIP allows for the quantitation of protein binding enrichment at specific genomic regions, thereby providing a new window into chromatin organization and gene regulation. This method employs chemical crosslinking to crosslink (or ‘fix’) DNA-protein interactions, and the chromatin is subsequently sheared into small fragments using enzymatic or physical methods. The DNA fragments are subjected to immunoprecipitation with antibodies against proteins of interest that bind directly or indirectly to genomic DNA. After immunoprecipitation, the DNA is released from its interacting proteins and analyzed for enrichment by qPCR. Although ChIP-qPCR is widely used, it still remains challenging and time intensive, in part due to the process of developing optimal PCR primers. More recently, drawing a genome-wide picture of protein-DNA interactions has been made possible through the development of ChIP followed by next generation sequencing (ChIP-seq) (20–22). Although this method is very powerful, it has not supplanted the need for ChIP-qPCR, which plays a crucial role in quality control before sequencing and validation of ChIP-seq findings. In addition, ChIP-qPCR is still used for focused experiments and verification of ChIP-seq findings.

Designing primers for ChIP-qPCR is significantly more challenging than for other qPCR methods for several reasons: (i) ChIP primers must target very specific regions, thereby limiting the options for primer design. This is particularly relevant for proteins with narrow binding regions, like transcription factors, where primers verified to work for one antibody in a promoter region might not work efficiently in another ChIP experiment. (ii) The quality of the DNA is reduced by the mechanical shearing as well as the chemical crosslinking. (iii) The quantity of available DNA is typically low: frequently < 5 ng. (iv) Intron-spanning primers are used to enhance specificity in qRT-PCR reactions, but as the template in ChIP is genomic DNA, this is not possible.

Even though ChIP primer information is provided in the materials and methods section of publications, it remains very time consuming to find suitable primers in the literature. Many publications describe ChIP-qPCR results with mathematical significance, however, the actual fold-change or the controls used prevent a determination of the general suitability of these primers. Alternate or outdated gene names in some publications constitute another difficulty. Despite the challenges in designing ChIP-qPCR primers, to the best of our knowledge, there is no ChIP primer repository/database available thus far. The current lack of a ChIP primer database can be attributed to the complexity of extracting high quality information of ChIP experiments from publications—a process that cannot be automated in a meaningful way. To address this need, we describe herein a database for published and verified ChIP-qPCR primer sequences, curated by manual screening, providing researchers a user-friendly interface to access and compare ChIP-qPCR primers, and get specifics on the experimental conditions in which the primers were used. The database is linked at https://umiamihealth.org/bascom-palmer-eye-institute/research/clinical-and-laboratory-research/ocular-oncology-laboratory/chip-primers and hosted at https://www.chipprimers.com/.

## MATERIALS AND METHODS

PubMed searches were performed using the search terms ‘ChIP-seq’, ‘ChIP-qPCR’ and ‘Chromatin Immunoprecipitation’ and publications were manually screened for ChIP-qPCR experiments. ChIP-qPCR primers were included when the experimental protocol and primer description were not ambiguous and study showed qPCR enrichment over a valid control of at least 5-fold. Gene names were verified with the NCBI database (https://www.ncbi.nlm.nih.gov/gene) for correct nomenclature. In cases where old gene names or aliases had been used, the gene name was replaced by the correct name and a note was made in the database entry stating the original gene name used by the authors. Further, the complete gene name (‘Description’) was added, alongside the antibody used, the species, and the cell line or tissue which was used in the experiment. If the authors reported enrichment only when cells or animals that were not wild type (e.g. treated, stimulated, gene knockdown), we stated the conditions in the notes of the respective database entry. The PubMed ID was included and is displayed as a direct clickable link in the user interface opening a new browser window leading to the original publication on the NCBI website. The melting temperature (*T*_m_) was calculated with OIigoCalc (23) assuming 50 nM primers and 50 mM salt (Na^+^). All entries were double-checked by two independent researchers.

## RESULTS

### Curating database entries of high quality primer sequences

A crucial part of determining ChIP enrichment at a genomic locus by qPCR is the use of valid controls. It is standard to perform PCR with control DNA in parallel, which can either be derived from an aliquot of the sonicated chromatin before the IP (‘input control’), or DNA from a mock ChIP performed with an IgG control antibody or an antibody specific for a non-nuclear antigen. Either of the two latter methods are accepted as valid controls as described in the *ENCODE ChIP-seq Guidelines* (24), and data is usually presented as ‘% of input’ after analysis. In addition, a primer set targeting a non-enriched region should be used. While some studies use a big change in the ‘% of input’ value to claim enrichment, it must be noted that without primers targeting non-enriched regions, a high ‘% of input’ value is technically not sufficient, even if other antibodies in the same experiment had lower ‘% of input’ values. The reason being that the ‘% of input’ value highly depends on the amount of DNA that is used in the PCR reactions, while antibodies, including control IgG antibodies, vary widely in their unspecific DNA pulldown. Hence, to curate a repository of high quality ChIP-qPCR primers, we manually screened publications for ChIP primers and only included primers that showed ≥5-fold enrichment over a valid control and a non-enriched region. If no non-enriched regions were used as controls, we only included primers in cases where a ≥5-fold enrichment was shown the same regions following different experimental conditions such as gene knockdown or drug treatment. While lower fold-changes may be biologically significant and are frequently reported in literature, a 5-fold cutoff increases the confidence and quality of the entries.

### Database structure

**Figure 1.**
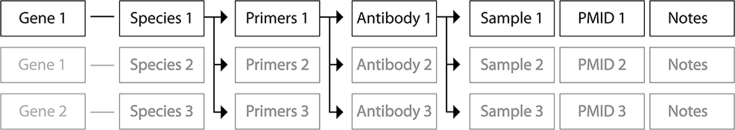
ChIPprimersDB Database structure. Each Gene/Species combination may have multiple primer entries, in turn allowing for multiple antibody entries. Each antibody may include multiple ChIP experiments, comprised of the sample used, the PMID as a clickable link, and Notes.

Each primary database entry consists of a gene name/species pair. All gene names were verified with the NCBI database for up-to-date names and abbreviations and corrected if incorrect or out-of-date. Each primary gene name/species entry may have multiple primer pair entries (primer sequences), which in turn can have multiple ‘Antibody’ entries. Each antibody may have multiple entries consisting of the precise sample used (which cell line or tissue), the PubMed ID of the publication (PMID), and notes on the experimental procedure (Figure 1) - *de facto* resembling individual ChIP experiments.

### Database interface and use

The interface of the database website by default displays all entries as a list, including the gene symbol, full gene name (‘Description’), and species. Further, each entry has a ‘Show Primers’ button, displaying the number of primer pairs available for the respective genes. By clicking the ‘Show Primers’ button, the database will display all primer sequences for the gene alongside their melting temperatures (*T*_m_), the antibodies used, the tissues or cell lines used (‘Sample’), the PubMed ID (PMID) of the publication reporting the primers, and ‘Notes’. The PMID is a clickable link which opens the PubMed website with the original article in a new browser window, to allow the user to quickly assess the complete information on the experimental setup and materials used for this ChIP experiment. The ‘Notes’ section contains additional information on the experiment, e.g. if cells were treated with drugs, a gene knockdown was performed, or alternate/outdated gene names were used in the manuscript.

**Figure 2.**
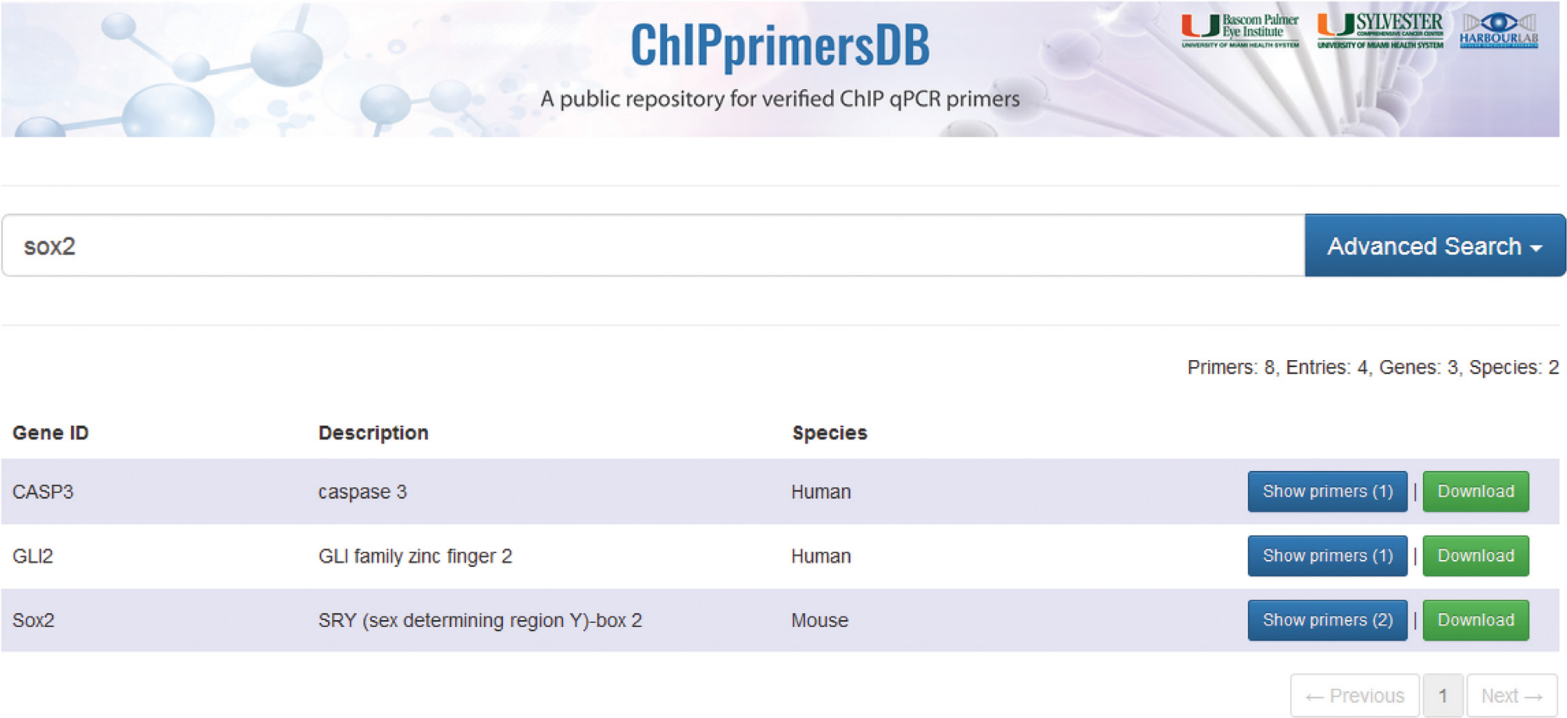
Example of a search performed on ChIPprimersDB. By default, the search function will search all database fields. A search for Sox2 results in three results, where one is the Sox2 gene entry containing primers for the Sox2 gene. The ‘Show primers’ button indicates that two different verified primer pairs are available for this gene. The genes CASP3 and GLI2 are listed in the search results, as those genes were found enriched in ChIP experiments for Sox2. Using the advanced search function, the user can limit the search to specific fields.

ChIPprimersDB has a search function that reacts in real time to the users input. By default, the search is performed for every field for each database entry (Figure 2). To allow more specific searches, we also implemented an ‘Advanced Search’ option to restrict the search to certain fields, allowing the user to search for e.g. genes or antibodies only. All entries can be easily downloaded by clicking on the ‘Download’ button, which provides a .csv file that can be viewed instantly for instance in Microsoft Excel. To enable users to submit their own published primer sequences, we also designed a submission form to streamline this process. Users can easily enter all the required information and submit the entry for revision by a site administrator, which will check if all quality measures outlined above are met. This will allow this repository to grow organically and for users to get credit for designing quality ChIP primers by citations from the database users.

## DISCUSSION

ChIPprimersDB serves multiple use cases frequently emerging when planning ChIP experiments: The direct link to the original publications on the PubMed website allows users to quickly compare the data from different groups and decide on an experimental strategy and a specific antibody, which yielded the best results. Choosing the right antibody is especially important, as there are significant differences in how antibodies perform in ChIP experiments, with only a fraction performing well. Further, searches for a specific antibody allow the users to pick primers for genes that can be used as positive controls to verify successful ChIP experiments. Another classic use case is to retrieve primers for genes of interest. Even if the database does not have primers for certain genes for the same antibody intended for use, the primers listed for individual gene regions are a good starting point and will perform well in many cases.

The web interface of ChIPprimersDB offers researchers a fast and convenient way to search for quality ChIP-primer sequences, compare published datasets and also submit own primer sequences to foster continuous growth. Together, this repository serves as an important tool for researchers planning ChIP experiments.
